# Hhatl ameliorates endoplasmic reticulum stress through autophagy by associating with LC3

**DOI:** 10.1016/j.jbc.2024.107335

**Published:** 2024-05-04

**Authors:** Xingjuan Shi, Jiayu Yao, Yexi Huang, Yushan Wang, Xuan Jiang, Ziwen Wang, Mingming Zhang, Yu Zhang, Xiangdong Liu

**Affiliations:** School of Life Science and Technology, Key Laboratory of Developmental Genes and Human Disease, Southeast University, Nanjing, China

**Keywords:** Hhatl, ER stress, autophagy, LC3, apoptosis

## Abstract

Endoplasmic reticulum (ER) stress, a common cellular stress response induced by various factors that interfere with cellular homeostasis, may trigger cell apoptosis. Autophagy is an important and conserved mechanism for eliminating aggregated proteins and maintaining protein stability of cells, which is closely associated with ER stress and ER stress–induced apoptosis. In this paper, we report for the first time that Hhatl, an ER-resident protein, is downregulated in response to ER stress. Hhatl overexpression alleviated ER stress and ER stress induced apoptosis in cells treated with tunicamycin or thapsigargin, whereas Hhatl knockdown exacerbated ER stress and apoptosis. Further study showed that Hhatl attenuates ER stress by promoting autophagic flux. Mechanistically, we found that Hhatl promotes autophagy by associating with autophagic protein LC3 (microtubule-associated protein 1A/1B-light chain 3) *via* the conserved LC3-interacting region motif. Noticeably, the LC3-interacting region motif was essential for Hhatl-regulated promotion of autophagy and reduction of ER stress. These findings demonstrate that Hhatl ameliorates ER stress *via* autophagy activation by interacting with LC3, thereby alleviating cellular pressure. The study indicates that pharmacological or genetic regulation of Hhatl-autophagy signaling might be potential for mediating ER stress and related diseases.

The endoplasmic reticulum (ER) represents a complex membranous network that plays multiple roles in the cell including protein synthesis and maturation, calcium storage, and lipid metabolism (1). The ER is a multifunctional organelle composed of many enzymes and chaperons that assist protein folding and posttranslation modifications, ensuring the maintenance of ER homeostasis. Physiological, chemical, or pathological factors that compromise ER homeostasis lead to the accumulation of unfolded or misfolded proteins in the ER lumen, which is known as ER stress. In response to ER stress, unfolded protein response (UPR) is triggered to reduce protein overload, promote the chaperon-regulated folding ability, and ultimately enhance unfolded protein degradation *via* autophagy and ER-associated degradation (2, 3). The UPR is transduced by three ER-resident sensors, inositol-requiring enzyme 1, activating transcription factor 6, and dsRNA-activated protein kinase–like ER kinase (4). Although UPR activation aims to restore ER homeostasis, persistent ER stress can activate apoptotic signals and damage the target cells, which also contribute to various metabolic and age-related disorders (5).

Autophagy is an evolutionarily conserved degradation system in which the intracellular contents are engulfed in autophagosomes, double-membrane vesicles, and then degraded in a lysosome-dependent manner (6). A series of autophagy-related gene (ATG) products participate in the induction and execution of autophagy. The processes of autophagosome elongation include the formation of ATG12–ATG5–ATG16L1 complex and the binding of lipid phosphatidylethanolamine to LC3 under the assistance of ATG4, ATG7, and ATG3 (7). Autophagy serves an essential role in restoring cellular homeostasis and maintaining cell survival under stress (8). Autophagy, closely related to ER stress, eliminates excessive proteins and acts as a mechanism of cell self-protection (9). It has been reported that ER stress may trigger autophagy by protein kinase–like ER kinase-eIF2α signaling or transcriptional activation of autophagic proteins mediated by CHOP (10, 11). It is also demonstrated that ER stress signaling activates autophagy by restraining mechanistic target of rapamycin kinase complex 1 and promoting beclin-1 and ATG expression (12). However, the signaling pathway coupling ER stress with autophagy is not fully understood.

*HHATL* (hedgehog acyltransferase-like), originally characterized as novel heart-specific gene, has been reported to play various biological function in different species (13). It is reported that Hhat (Hedgehog acyltransferase) is responsible for N-Palmitoylation of Sonic Hedgehog (14). Intriguingly, mammalian Gup1, known as Hhatl, has been reported to act as a negative regulator for N-terminal palmitoylation of Sonic hedgehog (15). In *Saccharomyces cerevisiae*, Gup1 participated in programmed cell death regulation (16). Our recent study showed that zebrafish *hhatla*, homolog of *Hhatl*, is involved in cardiac hypertrophy (17). However, the biological function of Hhatl in mammalians remains unclear. *Hhatl (Mg56)*-KO mice exhibited a suckling defect and died under starvation conditions due to abnormal postnatal muscle maturation (18). Mechanistic study demonstrated that the UPR response is severely activated in the knockout muscle, indicating that Hhatl deficiency might activate ER stress–induced UPR and disrupt postnatal development of skeletal muscle. However, the precise role of Hhatl in ER stress is still unclear. In this paper, we studied the function of Hhatl in ER stress in mammalian cells and provide the first evidence that Hhatl ameliorates ER stress by promoting autophagy and associating with LC3 protein.

## Results

### Hhatl expression is reduced under ER stress

**Figure 1:**
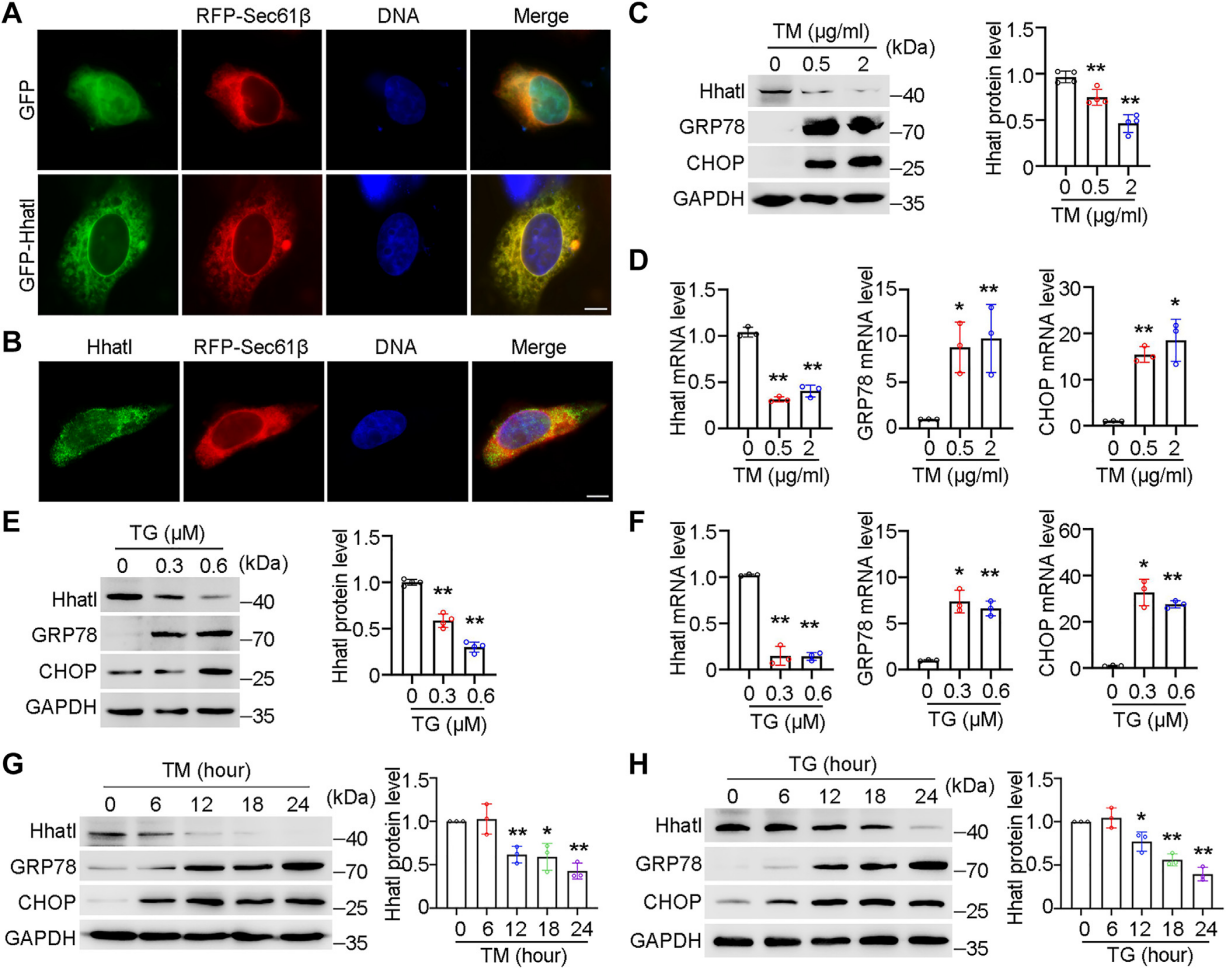
**Hhatl expression is reduced under ER stress.***A*, representative image of HeLa cells cotransfected with RFP-Sec61β and GFP or GFP-Hhatl followed by staining with DAPI (*blue*). Scale bar represents 10 μm. *B*, HeLa cells transfected with RFP-Sec61β and then stained with anti-Hhatl and the DNA dye DAPI. Scale bar represents 10 μm. *C*, HeLa cells were treated with gradient dosages of tunicamycin (TM) for 24 h, and expression levels of Hhatl, GRP78, and GAPDH were determined by Western blot. Densitometric quantification of the expression of Hhatl and GRP78 were analyzed. *D*, the relative mRNA levels of Hhatl, GRP78, and CHOP were examined in HeLa cells treated with gradient dosages of TM for 24 h. *E*, HeLa cells were treated with gradient dosages of thapsigargin (TG) for 24 h, and expression levels of Hhatl, CHOP, and GAPDH were examined by Western blot. Densitometric quantification of the expression of Hhatl and CHOP were analyzed. *F*, the relative mRNA levels of Hhatl, GRP78, and CHOP were examined in HeLa cells treated with gradient dosages of TG for 24 h. *G* and *H*, HeLa cells were treated with TM (2 μg/ml) (*G*) or TG (0.6 μM) (*H*) for different times, and expression levels of Hhatl, CHOP, and GAPDH were examined by Western blot. Densitometric quantification of Hhatl expression was analyzed. Data are presented as means ± SD (*n* = 3). One-way ANOVA, ∗*p* < 0.05, ∗∗*p* < 0.01. ER, endoplasmic reticulum; TG, thapsigargin; TM, tunicamycin.

Previous study has demonstrated that Hhatl is a sarcoplasmic reticulum–localized protein in mouse skeletal muscle (18). To characterize the subcellular localization of Hhatl, we transfected HeLa cells with RFP-Sec61β, a frequently used ER marker and GFP-Hhatl. By immunofluorescence microscopy, we found that Hhatl colocalized with Sec61β, which indicated that Hhatl localized at ER (Fig. 1*A*). Besides, the immunostaining of endogenous Hhatl showed the colocalization of Hhatl and ER marker Sec61β (Fig. 1*B*). To explore whether Hhatl participates in ER stress, we first treated cells with an ER stress inducer tunicamycin (TM). Compared with control group, cells treated with TM exhibited elevated expression of GRP78 and CHOP, representative markers of ER stress, and significantly reduced Hhatl protein level (Fig. 1*C*). Besides, TM treatment contributed to strikingly elevated mRNA level of GRP78 and CHOP, as well as decreased mRNA level of Hhatl (Fig. 1*D*). We further administered another ER stress activator thapsigargin (TG) into the cells and consistently found that ER stress contributed to the dramatically decreased Hhatl expression accompanied by elevated levels of GRP78 and CHOP (Fig. 1, *E* and *F*). To check whether the downregulation of Hhatl under ER stress is time-dependent, HeLa cells were treated with TM or TG with different times. We found that the expression of Hhatl is gradually reduced with prolonged treatment of TM or TG (Fig. 1, *G* and *H*).

To verify above result, we further checked the RNAseq studies for ER stress *via* GEO database. We found that the Hhatl expression is significantly downregulated in SH-SY5Y human neuroblastoma cells treated with TG (*p* = 1.964E-03) or TM (*p* = 3.685E-04) compared with control group (GSE24497) (Table S1). We further investigated whether the expression of Hhatl is regulated by physiological ER stress–like protein overload. The null Hong Kong variant of a-1-antitrypsin (NHK), a secretion-incompetent variant of a-1-antitrypsin, is a misfolded substrate for ER-associated degradation (19, 20). The physiological ER stress was induced by transfecting cells with pcDNA3.1-HA-NHK. As shown in Fig. S1, the expression of Hhatl is markedly decreased in cells transfected with HA-NHK, indicating that Hhatl is downregulated under physiological ER stress. These results demonstrate that ER stress downregulates Hhatl expression.

### Hhatl alleviates TM or TG-induced ER stress

**Figure 2:**
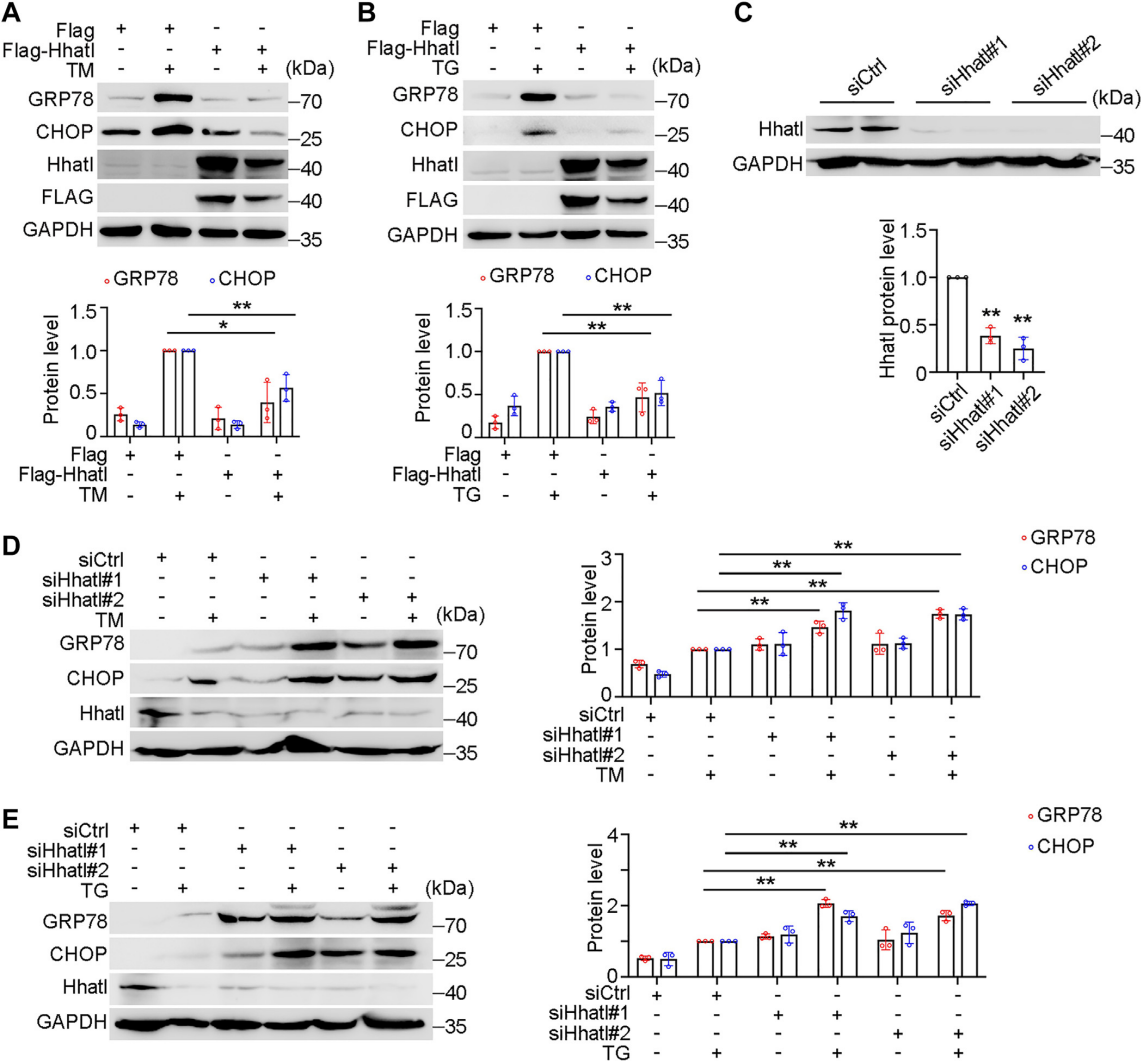
**HHATL gain of function is sufficient to alleviate ER stress.***A* and *B*, HeLa cells were transfected with Flag or Flag-Hhatl and then treated with DMSO or TM (2 μg/ml) (*A*) or TG (0.6 μM) (*B*) for 24 h. Cell lysates were subjected to immunoblotting and probed with the indicated antibodies. Densitometric quantification for the expression of GRP78 or CHOP was normalized to GAPDH protein levels. *C*, cells were transfected with control or Hhatl siRNAs, and the levels of Hhatl and GAPDH were then examined by immunoblotting. Densitometric quantification for the expression of Hhatl was normalized to GAPDH protein level. *D* and *E*, cells were transfected with control or Hhatl siRNAs for 72 h, and TM (*D*) or TG (*E*) were added 24 h before harvesting. Densitometric quantification for the expression of GRP78 or CHOP was normalized to GAPDH protein levels. Data are presented as means ± SD (*n* = 3). Student’s *t* test and One-way ANOVA, ∗*p* < 0.05, ∗∗*p* < 0.01. ER, endoplasmic reticulum; TG, thapsigargin; TM, tunicamycin.

To explore the function of Hhatl in ER stress, HeLa cells were transiently transfected with Flag vector or Flag-Hhatl and treated with TM or TG. Compared with control group, Hhatl strikingly lowered protein levels of GRP78 and CHOP upon TM or TG administration, indicating a decrease in ER stress (Fig. 2, *A* and *B*). Given the role of Hhatl in ameliorating ER stress, we speculated that Hhatl deficiency may aggravate ER stress. To test this hypothesis, we further transfected cells with control or Hhatl siRNAs and collected cells for immunoblotting. Compared with control group, Hhatl siRNAs dramatically reduced Hhatl expression (Fig. 2*C*). We treated siRNA-transfected cells with TM or TG and examined the ER stress markers. Compared with control group, the elevated protein levels of GRP78 and CHOP were detected in Hhatl-depleted cells (Fig. 2, *D* and *E*). These results indicate that Hhatl depletion aggravates drug-induced ER stress.

### Hhatl deficiency exacerbates ER stress–induced apoptosis

**Figure 3:**
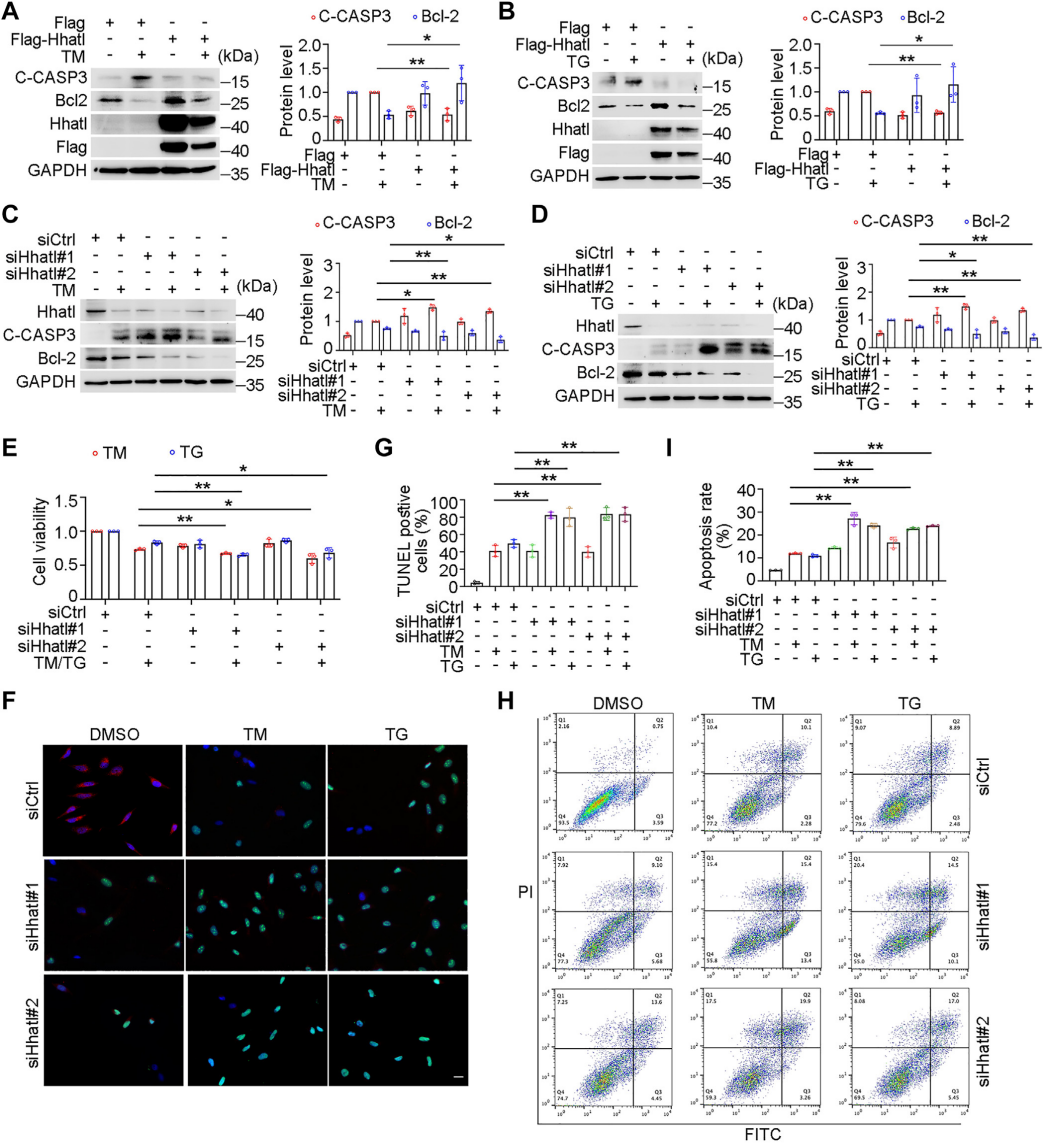
**Hhatl deficiency exacerbates ER stress–induced apoptosis.***A* and *B*, HeLa cells were transfected with Flag or Flag-Hhatl and then treated with DMSO or TM (*A*) or TG (*B*) for 24 h. Cell lysates were subjected to immunoblotting, and the protein levels of cleaved-caspase 3 (C-CASP3), Bcl2, and Hhatl were examined. *C*–*E*, cells were transfected with control or Hhatl siRNAs and then treated with DMSO, TM, or TG. Cell apoptosis and viability was measured separately by Western blot (*C* and *D*) and cell counting kit-8 assay (*E*). *F* and *G*, cells were transfected with control or Hhatl siRNAs and then treated with DMSO, TM, or TG. Cells were fixed and successively stained with TUNEL BrightGreen apoptosis detection kit (*green*), anti-Hhatl antibody (*red*), and DNAI (*blue*) (*F*). Scale bar represents 10 μm. The TUNEL-positive cells were calculated (*G*). *H* and *I*, cells were transfected with siRNAs and treated with DMSO, TM, or TG. Cells were then stained with Annexin V-FITC/PI Apoptosis Detection Kit, and the proportion of apoptosis was detected by flow cytometry analysis (*H*). The apoptosis rate of cells was measured (*I*). Data are presented as means ± SD (*n* = 3). One-way ANOVA, ∗*p* < 0.05, ∗∗*p* < 0.01. ER, endoplasmic reticulum; TG, thapsigargin; TM, tunicamycin.

It has been established that persistent ER stress might contribute to apoptosis (21). We then investigated the role of Hhatl in ER stress–triggered apoptosis by transfecting cells with Flag or Flag-Hhatl. TM or TG treatment induced the protein level of apoptosis indicator cleaved-caspase 3 (C-CASP3) and reduced the level of anti-apoptotic protein Bcl2, whereas Hhatl restored the expression of C-CASP3 and Bcl2 in response to ER stress (Fig. 3, *A* and *B*). We further examined whether Hhatl deficiency aggravated ER stress–induced apoptosis by knocking down Hhatl *via* RNA interference. Consistently, Hhatl depletion further promoted the increased level of C-CASP3 and lowered the decreased level of Bcl-2 during ER stress (Fig. 3, *C* and *D*). Moreover, Hhatl deficiency inhibited cell survival upon ER stress (Fig. 3*E*). To further visualize the cell apoptosis, we performed TUNEL assay analysis. Compared with the control group, Hhatl depletion increased the percentage of TUNEL-positive apoptotic cells under ER stress (Fig. 3, *F* and *G*). Besides, flow cytometry analysis detected that the proportion of apoptosis in Hhatl knockdown cells is significantly elevated (Fig. 3, *H* and *I*). These data demonstrate that Hhatl deficiency aggravates ER stress–induced apoptosis.

### Hhatl regulates autophagy activation under ER stress

**Figure 4:**
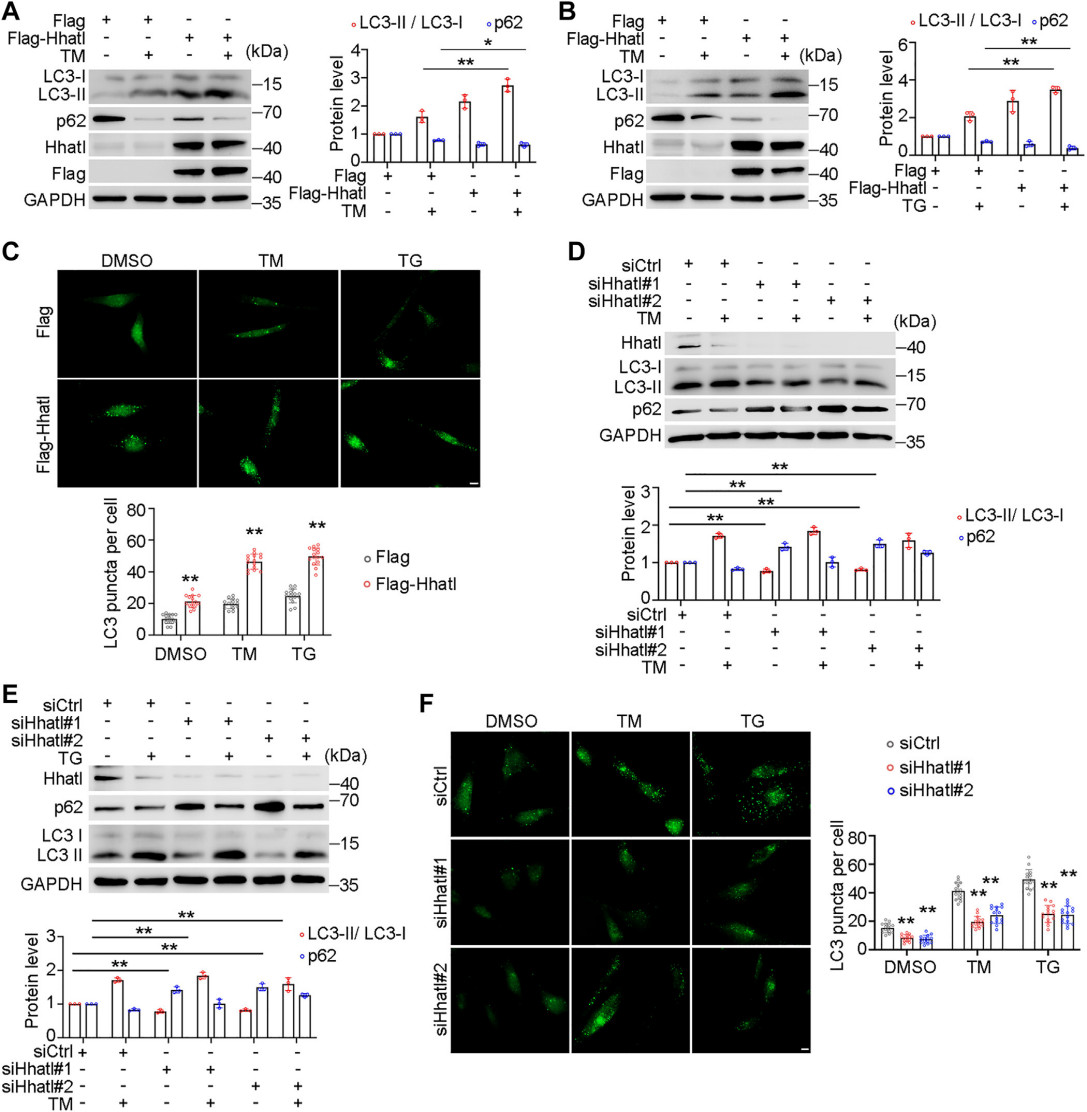
**Hhatl regulates autophagy activity under ER stress.***A* and *B*, HeLa cells were transfected with Flag or Flag-Hhatl and then treated with DMSO or TM (*A*) or TG (*B*). Cell lysates were subjected to immunoblotting and probed with the indicated antibodies. *C*, HeLa cells transfected with Flag or Flag-Hhatl were treated with DMSO, TM, or TG and then followed by anti-LC3 antibody staining. The LC3 puncta were detected and analyzed. Scale bar represents 10 μm. *D* and *E*, cells were transfected with control or Hhatl siRNAs for 72 h, and TM (*D*) or TG (*E*) were added 18 h before harvesting. The protein level of LC3-II and GAPDH were examined by immunoblotting. *F*, cells were cotransfected with control or Hhatl siRNAs, and TM or TG were added 18 h before cell fixation. Cells were then stained with anti-LC3 antibody and LC3 puncta were examined under the fluorescence microscope. Scale bar represents 10 μm. Data are presented as means ± SD (*n* = 3). One-way ANOVA, ∗*p* < 0.05, ∗∗*p* < 0.01. ER, endoplasmic reticulum; TG, thapsigargin; TM, tunicamycin.

It is reported that ER stress triggers autophagy to remove excessive protein and protect cells from damage or stress (22). During autophagy activation, the cytosolic form of LC3 (LC3-I) transforms to the lipid form (LC3-II). We found that Hhatl overexpression dramatically elevated LC3-II expression under ER stress triggered by TM or TG (Fig. 4, *A* and *B*). In addition to ER stress, the initiation of autophagy can be induced by other stress conditions, such as nutrient starvation and energy deprivation (23). Immunoblot assays indicated that Hhatl protein expression was reduced in nutrient-starved cells treated with Earle’s balanced salt solution (Fig. S2*A*). Consistently, Hhatl strikingly promoted LC3-II expression under starvation, indicating that Hhatl promotes starvation-induced autophagy (Fig. S2*B*). Immunofluorescence analysis demonstrated more endogenous LC3 puncta in the cells with Hhatl overexpression, suggesting that Hhatl enhanced activation of autophagy (Fig. 4*C*). Conversely, depletion of Hhatl strikingly reduced LC3-II expression under ER stress (Fig. 4, *D* and *E*). Compared with control group, reduced LC3 puncta was observed in cells with Hhatl depletion (Fig. 4*F*). These data display that Hhatl enhances the activation of autophagy in response to ER stress.

### Hhatl protects cells from ER stress by promoting autophagy

**Figure 5:**
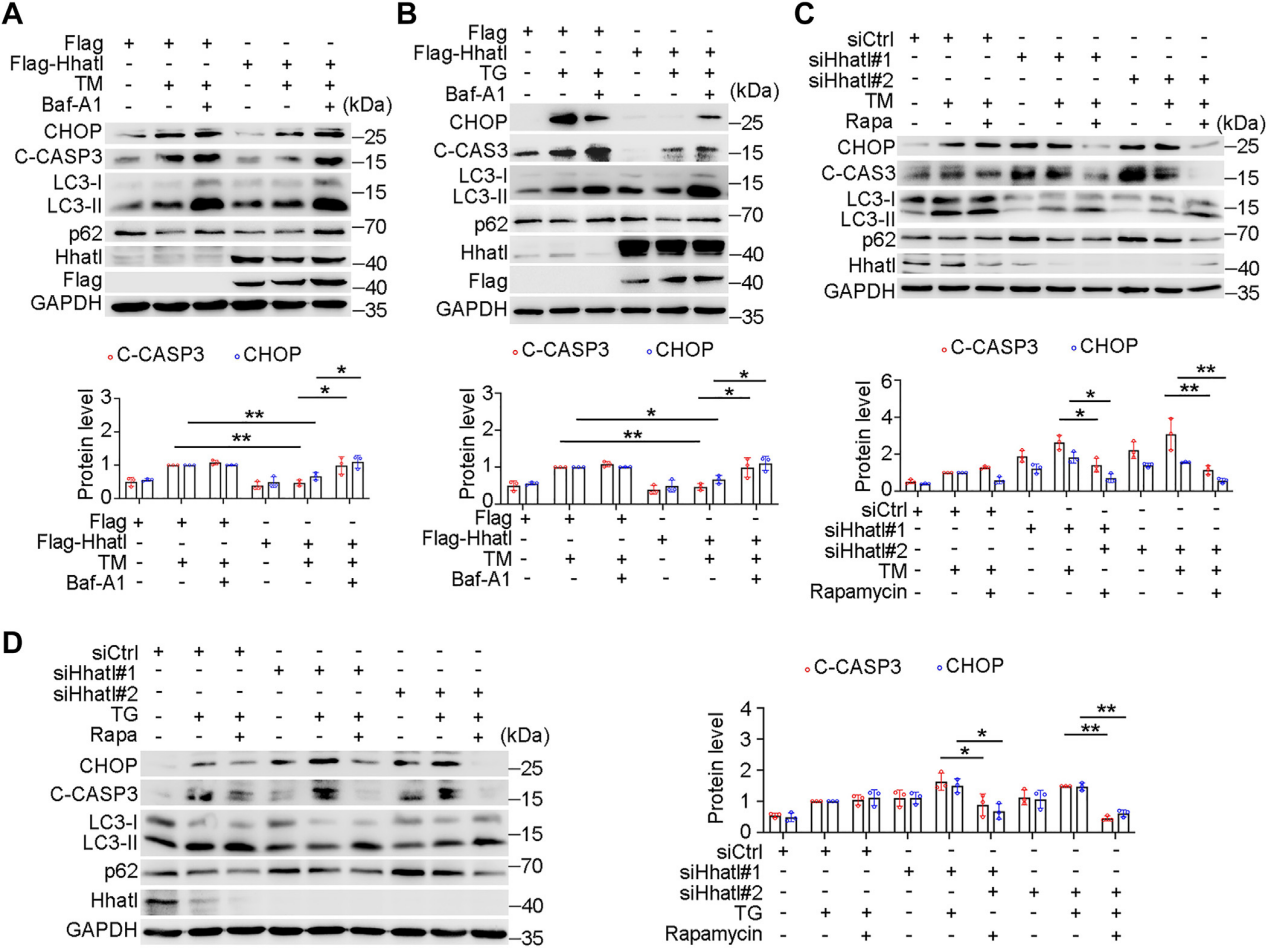
**Hhatl protects cells from ER stress by promoting autophagy.***A* and *B*, cells were transfected with Flag or Flag-Hhatl, treated with DMSO or TM (*A*) or TG (*B*), and 400 nM Bafilomycin A1 (Baf-A1) was added 16 h before cell harvesting. The protein levels of CHOP, cleaved caspase 3 (C-CASP3), LC3-II, and p62 were examined by immunoblotting. *C* and *D*, cells were transfected with control or Hhatl siRNAs for 72 h, treated with TM (*C*) or TG (*D*), and 1 μM Rapamycin (Rapa) was added 16 h before cell harvesting. Cell lysates were subjected to immunoblotting and probed with the indicated antibodies. Data are presented as means ± SD (*n* = 3). One-way ANOVA, ∗*p* < 0.05, ∗∗*p* < 0.01. ER, endoplasmic reticulum; TG, thapsigargin; TM, tunicamycin.

To verify whether autophagy participates in Hhatl-mediated ER stress, we first analyzed the ER stress as well as anti-apoptotic biomarkers by first adopting bafilomycin A1 (Baf-A1), an inhibitor of vacuolar-type H^+^-ATPase affecting the pH of lysosomes. Baf-A1 has been widely used as an autophagy inhibitor by blocking autophagosome-lysosome fusion and inhibiting acidification and protein degradation in lysosomes. Treatment with Baf-A1 abolished the protective role of Hhatl in ER stress induced by TM or TG, evidenced by the increased level of CHOP and C-CASP3 (Fig. 5, *A* and *B*). Conversely, treatment of rapamycin, an autophagy activator, eliminated the pro-apoptotic effect of Hhatl depletion upon ER stress, as shown by reduced level of CHOP and C-CASP3 (Fig. 5, *C* and *D*). These result shows that Hhatl protects cells from ER stress by promoting autophagy.

### Hhatl interacts with the autophagic protein LC3 *via* the LIR motif

**Figure 6:**
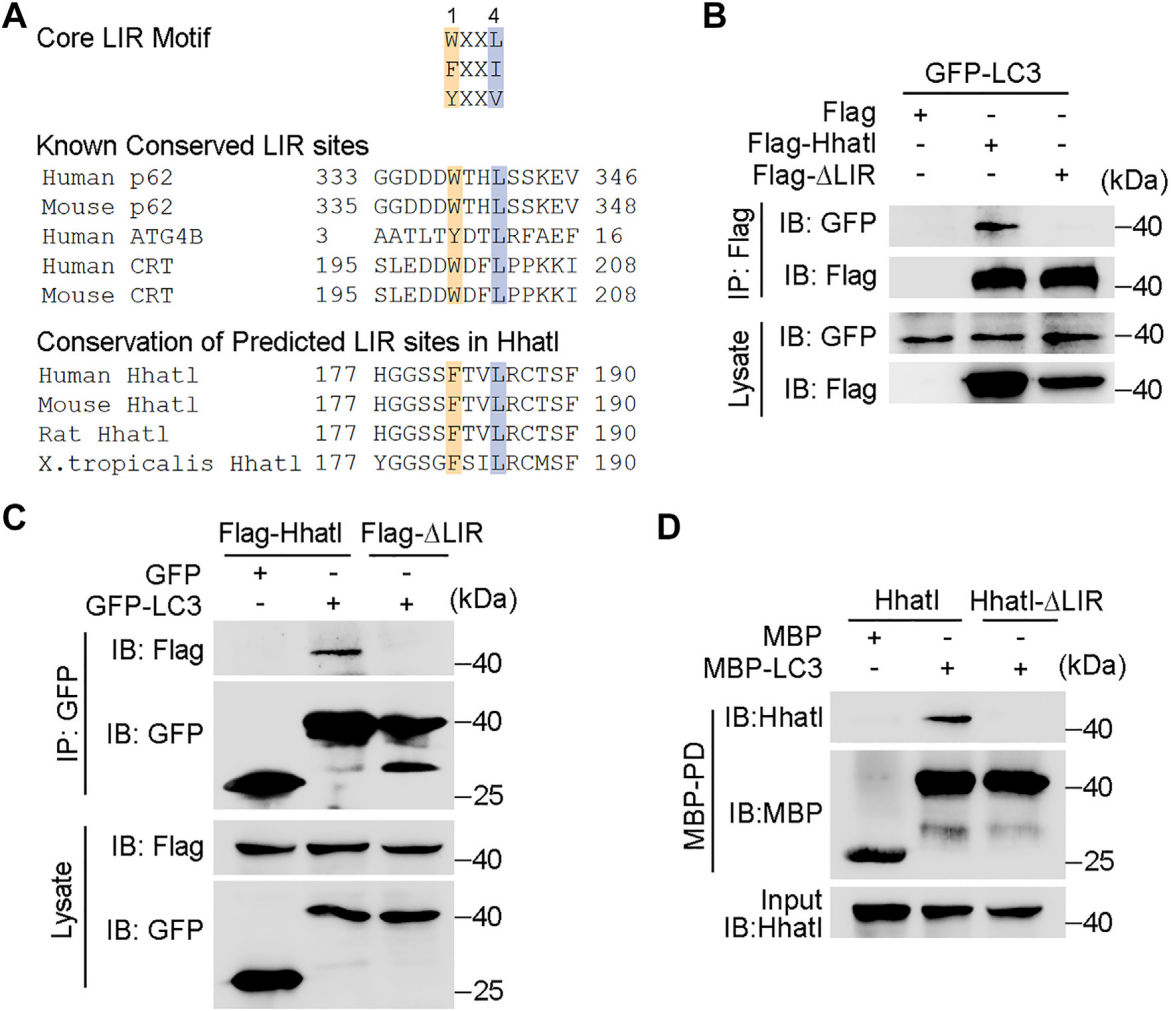
**Hhatl physically interacts with the autophagic protein LC3 through the LIR motif.***A*, alignment of the LIR. *B*, HEK293T cells were cotransfected with GFP-LC3 and Flag, Flag-Hhatl, or Flag-ΔLIR, and cell lysates were subjected to immunoprecipitation with an anti-Flag antibody and probed with the indicated antibodies. *C*, HEK293T cells were cotransfected with Flag-Hhatl or Flag-ΔLIR and GFP or GFP-LC3, and anti-GFP immunoprecipitates were probed with the indicated antibodies. *D*, *in vitro*–translated Hhatl or Hhatl-ΔLIR was incubated with bacterially purified MBP or MBP-LC3 immobilized on agarose beads. The presence of Hhatl in the MBP pull-down preparation was examined by immunoblotting. LIR, LC3-interacting region.

To gain deep insight into the relationship between Hhatl and the autophagy, we explored the association between Hhatl and autophagic proteins. It is known that the conserved LC3-interacting region (LIR) containing the motif W/Y/FXXL/I/V (X represents any residue) exists in most LC3 interaction proteins (24). Intriguingly, a conserved LIR motif is found in human Hhatl protein from amino acids 182 to 185 through iLIR autophagy database (Fig. 6*A*) (24, 25). To explore the interaction of Hhatl with LC3, we transfected HEK293T cells with Flag-Hhatl and GFP-LC3 and performed immunoprecipitation assays. As shown in Figure 6*B*, GFP-LC3 was precipitated with an anti-Flag antibody, indicating an interaction between Flag-Hhatl and GFP-LC3. In addition, immunoprecipitation assays also revealed an interaction between endogenous Hhatl and endogenous LC3 in cells (Fig. S3*A*). We further examined whether the binding capacity of Hhatl with LC3 might be altered under ER stress. To avoid possible influence of alterations of endogenous protein expression under stress conditions, Flag-Hhatl and GST-LC3 were overexpressed in HEK293T cells, and then immunoprecipitation assay was performed. We found that the association between Hhatl and LC3 was profoundly decreased in response to TM or TG treatment (Fig. S3*B*).

To verify the binding site of Hhatl, the null mutation of the LIR motif (F182A, L185A) of Hhatl (Hhatl ΔLIR) was established and subjected to immunoprecipitation. Markedly, the Hhatl ΔLIR mutant did not associate with GFP-tagged LC3 (Fig. 6*B*). Conversely, Flag-tagged Hhatl rather than Hhatl ΔLIR mutant was precipitated with an anti-GFP antibody, indicating that the interaction between Hhatl and LC3 is mediated by LIR motif (Fig. 6*C*). To study whether Hhatl and LC3 interact directly, *in vitro*–translated Hhatl or Hhatl ΔLIR mutant was incubated with bacterially purified MBP or MBP-LC3. Immunoblot analysis showed that Hhatl is present in MBP-LC3 pull-down preparation (Fig. 6*D*), suggesting a direct interaction between Hhatl and LC3.

### The interaction between Hhatl and LC3 is indispensable for autophagy activation and ER stress reduction regulated by Hhatl

**Figure 7:**
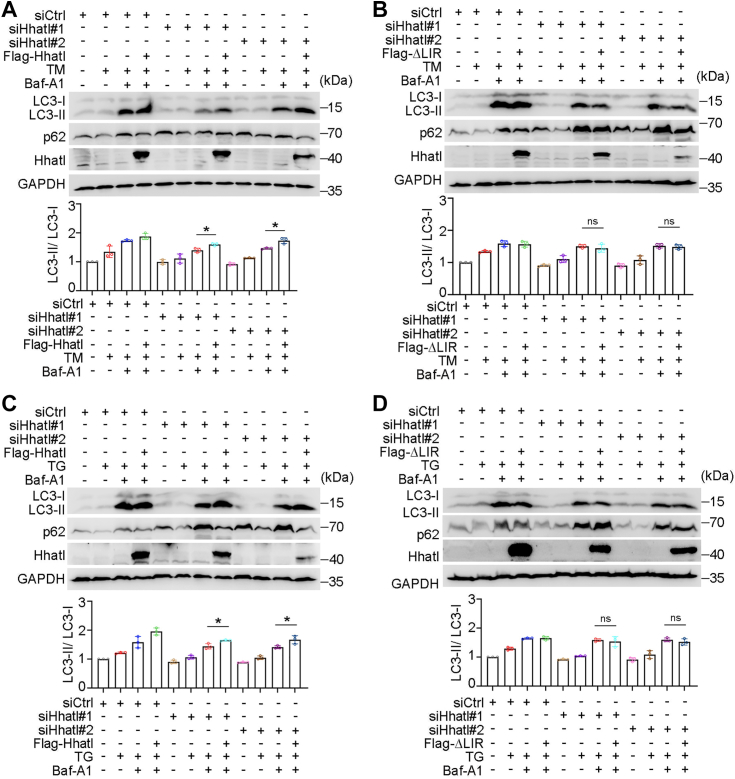
**The interaction between Hhatl and LC3 is required for Hhatl-mediated promotion of autophagy.***A* and *B*, HeLa cells were first transfected with control or Hhatl siRNAs for 48 h and then transfected with Flag-Hhatl (*A*) or Flag-ΔLIR mutant (*B*) and incubated with TM and without or with Bafilomycin A1. Cell lysates were subjected to immunoblotting, and the expression level of LC3-II was analyzed. *C* and *D*, HeLa cells were first transfected with control or Hhatl siRNAs and then transfected with Flag-Hhatl (*C*) or Flag-ΔLIR mutant (*D*) and incubated with TG and without or with Bafilomycin A1. Cell lysates were subjected to immunoblotting, and the expression level of LC3-II was analyzed (*D*). Data are presented as means ± SD (*n* = 3). One-way ANOVA, ∗*p* < 0.05. LIR, LC3-interacting region; TM, tunicamycin; TG, thapsigargin.

To further study whether Hhatl–LC3 interaction participates in the modulation of autophagy, cells were first transfected with Hhatl siRNAs to deplete endogenous Hhatl, then transfected with Flag-Hhatl or Flag-ΔLIR mutant, and administered with TM or TG. We further performed autophagic flux assays using Baf-A1 to accumulate autophagosomal LC3-II. In contrast to Flag-Hhatl–expressing cells, Hhatl-induced expression of LC3-II was significantly abrogated in Hhatl-ΔLIR mutant expressing cells under TM-induced ER stress (Fig. 7, *A* and *B*). Consistently, Hhatl elevated level of LC3-II was largely abolished in Hhatl ΔLIR mutant expressing cells under ER stress triggered by TG, indicating a significant reduce in autophagic flux (Fig. 7, *C* and *D*). These data display that LIR motif is required for Hhatl in promoting autophagy.

**Figure 8:**
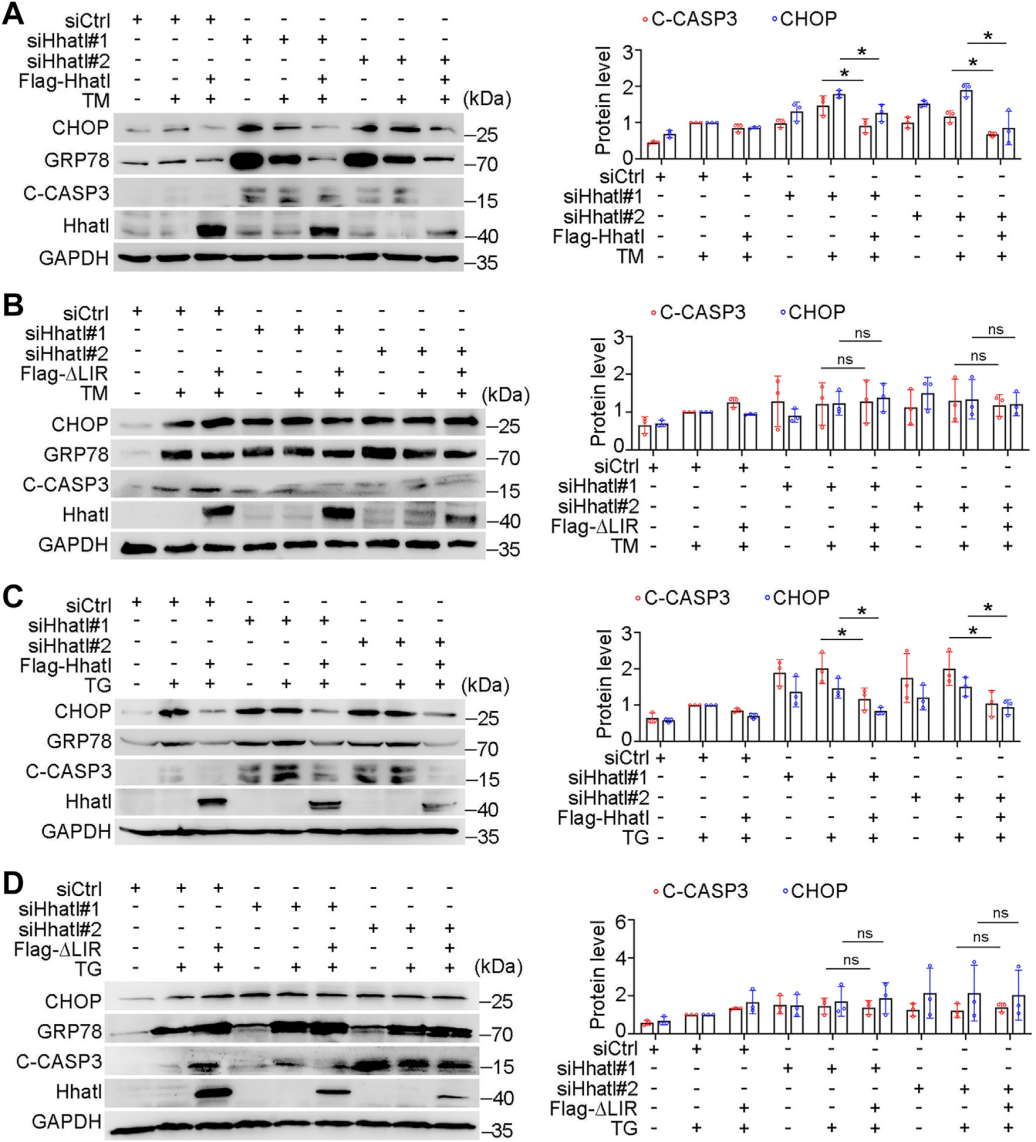
**The interaction between Hhatl and LC3 is required for Hhatl-mediated reduction of ER stress and apoptosis.***A* and *B*, cells were first transfected with control or Hhatl siRNAs for 48 h and then transfected with Flag-Hhatl (*A*) or Flag-ΔLIR mutant (*B*) and incubated with DMSO or TM. Cell lysates were subjected to immunoblot analysis and probed with the indicated antibodies for ER stress and apoptosis. Densitometric quantification of expression of C-CASP3 and CHOP were analyzed. *C* and *D*, cells were first transfected with control or Hhatl siRNAs for 48 h, and then transfected with Flag-Hhatl (*C*) or Flag-ΔLIR mutant (*D*) and incubated with DMSO or TG. Cell lysates were subjected to immunoblot analysis, and the protein expression of C-CASP3 and CHOP were analyzed. Data are presented as means ± SD (*n* = 3). One-way ANOVA, ∗*p* < 0.05. C-CASP3, cleaved-caspase 3; ER, endoplasmic reticulum; LIR, LC3-interacting region; TG, thapsigargin; TM, tunicamycin.

We then examined the ER stress levels in Hhatl ΔLIR-expressing cells compared with Hhatl WT-expressing cells treated with TM or TG. Hhatl induced attenuated level of CHOP and C-CASP3 was abrogated in Hhatl ΔLIR mutant expressing cells, suggesting that LIR motif is essential for the protective role of Hhatl in response to TM-induced ER stress (Fig. 8, *A* and *B*). Consistently, Hhatl reduced expression of ER stress and apoptosis biomarkers was markedly abolished in Hhatl-ΔLIR mutant expressing cells under ER stress triggered by TG (Fig. 8, *C* and *D*). These data demonstrate that the association between Hhatl and LC3 is indispensable for the protective role of Hhatl against ER stress.

## Discussion

In this study, we characterized that ER-localized protein Hhatl acts as a novel regulator in ER stress. In response to ER stress, the mRNA and protein levels Hhatl were both down-regulated. Intriguingly, Hhatl overexpression alleviated ER stress burden in cells, evidence by the reduced level of ER stress biomarkers GRP78 and CHOP. In contrast, knockdown of Hhatl aggravated stress burden in parallel experiments, indicating that Hhatl is essential to reverse the ER stress condition. It has been established that persistent ER stress might induce apoptosis (21). During TM- or TG-induced ER stress, the expression of endogenous Hhatl was decreased, and cells were more prone to apoptosis. We found that Hhatl overexpression contributes to cell survival undergoing ER stress. Conversely, knockdown of Hhatl promoted ER-induced apoptosis. Furthermore, the exogenous Flag-Hhatl markedly ameliorated the ER stress and apoptosis triggered by Hhatl depletion. These results demonstrate that Hhatl is a novel regulator of ER stress and serves a protective role under ER stress. It is worth noting that the decreased expression of Hhatl under physiological ER stress also indicates the significance of Hhatl in regulating ER stress. It is of interest to explore the UPR signaling pathway regulating the inhibited expression of Hhatl in further study.

We found that Hhatl enhances LC3-II levels in cells treated with ER stress inducers, indicating the elevated autophagic levels. Besides, treatment with autophagy inhibitor Baf-A1 eliminated the protective role of Hhatl in ER stress. Conversely, employment of autophagy activator rapamycin abolished the pro-apoptotic role of Hhatl depletion upon ER stress. These data indicate that Hhatl might trigger autophagy to ameliorate accumulation of misfolded proteins in ER stress, which is consistent with previous report that autophagy exerts a protective role in ER stress (22). LC3 is an important autophagy protein, which participates in the formation, elongation of autophagosome, and its fusion with lysosome (26). LC3 interacts with autophagy receptors through the evolutionarily conserved LIR motif and promotes target protein degradation (27, 28, 29). In this study, we report for the first time that Hhatl associates with LC3 *via* the LIR motif of Hhatl protein. Immunoprecipitation and MBP-pulldown analysis showed that Hhatl-ΔLIR, a mutant deficient in LIR motif, abolishes the interaction of Hhatl with LC3. Furthermore, Hhatl-ΔLIR overexpression abrogated Hhatl-mediated autophagic flux and failed to alleviate TM- or TG-induced ER stress and apoptosis. These data indicate that the LIR motif is crucial for the interaction of Hhatl and LC3 as well as the protective effect of Hhatl under ER stress.

**Figure 9:**
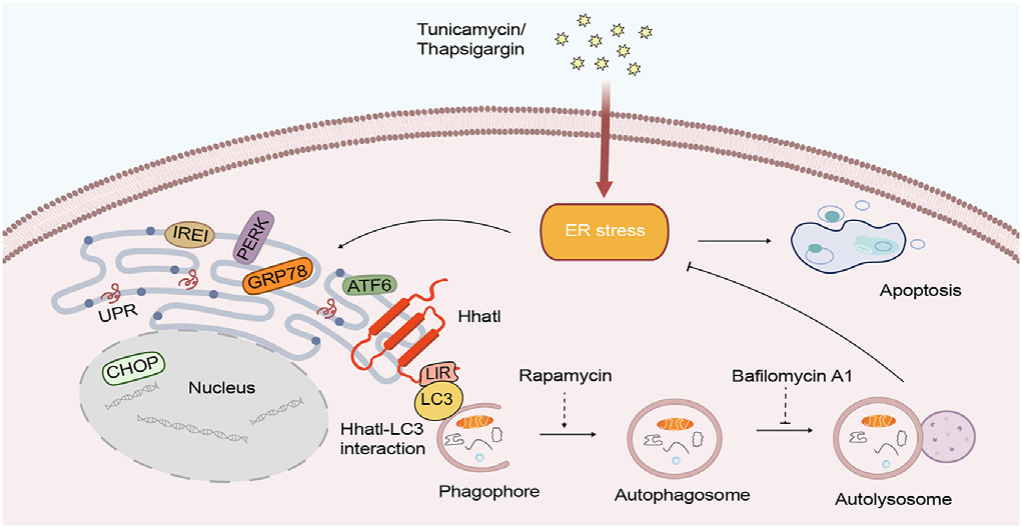
**Model of the role of Hhatl in ER stress.** Hhatl, an endoplasmic reticulum-resident protein, is significantly downregulated in response to ER stress. Hhatl protects cells from ER stress and ER stress-induced apoptosis by promoting autophagy. Treatment with autophagy inhibitor Bafilomycin A1 abolishes the protective role of Hhatl in ER stress. Conversely, treatment with autophagy activator rapamycin eliminates the pro-apoptotic effect of Hhatl depletion upon ER stress. Moreover, Hhatl physically interacts with the autophagic protein LC3 through the LIR motif. Mutation of the LIR motif eliminates Hhatl-mediated promotion of autophagy and reduction of ER stress. ER, endoplasmic reticulum; LIR, LC3-interacting region.

Taken together, we propose that Hhatl ameliorates ER stress through autophagy *via* associating with LC3 (Fig. 9). The interesting discovery of the interaction between Hhatl and LC3 provides novel insights into the function of Hhatl, linking ER stress with autophagy through interaction with degradation mechanism. Nevertheless, the underlying mechanisms by which Hhatl enhances autophagy activation require further investigation. It is possible that Hhatl promotes nuclear export of LC3 or facilitates the conversion of LC3-I to LC3-II, contributing to autophagic flux enhancement (30). Autophagy is a conserved catabolic recycling pathway in cells for surviving under multiple stress conditions. It has been reported that autophagy dysfunction is linked with the pathogenesis of major human disorders including cancer, cardiovascular, and musculoskeletal diseases (31, 32). The role of Hhatl in alleviating ER stress *via* autophagy might provide novel therapeutic strategy for diseases related with stress.

## Experimental procedures

### Reagents and antibodies

TM (T7765) and TG (T9033) were purchased from Sigma-Aldrich; Baf-A1 (S1413) and Rapamycin (Rapa, AY-22989) were purchased from Selleck. The Hhatl antibody was purchased from Sigma-Aldrich (67871104); LC3 antibody (2775) and CHOP antibody (2895) were from Cell Signaling Technology. The GRP78 antibody was from Santa Cruz Biotechnology (sc-13539), cleaved-caspase 3 antibody was from ABclonal (A11021), and GST antibody was from Abmart (M20007S). The p62 antibody (18420-1-AP), LC3 antibody (14600-1-AP), GAPDH antibody (60004-1-Ig), β-actin antibody (60008-1-Ig), GFP antibody (50430-2-AP), Flag antibody (20543-1-AP), Bcl-2 antibody (68103-1-Ig), and horseradish peroxidase-conjugated secondary antibodies (SA00001-1 and SA00001-2) were purchased from Proteintech.

### Plasmids, siRNA, and proteins

Flag-tagged Hhatl and Hhatl ΔLIR were constructed by cloning the human complementary DNA (cDNA) into into the pcDNA3.1-DYK mammalian vector as described previously (17). Human Hhatl ΔLIR plasmid in which the phenylalanine at the 182nd amino acid and leucine at the 185th amino acid were substituted with alanine (Phe^182^ to Ala^182^, Leu^185^ to Ala^185^) were constructed with Site-directed Mutagenesis Kit (Sangon Biotech, B639281). The following primers were used: Flag-Hhatl-ΔLIR: (forward: GGGGCAGCAGCGCCACAGTGGCGCGTTGCACCAGCTTTGCAC, reverse: CTGGTGCAACGCGCCACTGTGGCGCTGCTGCCCCCATGAAAC). Hhatl oligonucleotides were synthesized by Genscript, and the plasmids were sequenced by Sangon Biotech. Mammalian expression plasmids for GFP-LC3B, GST-LC3B were kindly provided by Professor Yu Li (33, 34), and plasmid of pcDNA3.1-HA-NHK was a kind gift from Professor Likun Wang in Chinese Academy of Sciences. Human Hhatl siRNA oligonucleotides (siHhatl#1: TCCGCTCCTGGATGTATGC; siHhatl#2: CCTCAACTTTGAGCTCTGG) were synthesized (GenPharma).

To purify MBP-LC3B, LC3B cDNA was cloned into the pMALp2T vector to generate the pMALp2T-LC3B plasmid. LC3B was expressed as a fusion to the C terminus of MBP. MBP-LC3B was purified with the amylose-conjugated Dextrin Beads according to the manufacturer’s instructions (Smart-Lifesciences, SA077005). *In vitro* transcription and translation of Hhatl were performed using the TNT SP6 high-yield wheat germ protein expression system (Promega) as described previously (35).

### Cell culture and transfection

HeLa and HEK293T cells were cultured in Dulbecco’s modified Eagle’s medium (Gibco, C11995500BT) supplemented with 10% fetal bovine serum (ExCell Bio, FSP500, VivaCell), 100 μg/ml penicillin, and 100 μg/ml streptomycin (New Cell & Molecular Biotech Co, C100C5) at 37 °C in a humidified incubator with 5% CO_2_. To perform transfection, cells were seeded on dishes or plates (Nest) to achieve an appropriate confluency without antibiotics. Plasmids were transfected into cells with PEI (Polysciences, 23966) or Lipo8000 (Beyotime, C0533). Hhatl siRNA oligonucleotides were transfected into cells with LipoRNAi transfection reagent (Beyotime, C0535) according to manufacturers’ protocols.

### RNA extraction and quantitative real-time PCR

Total RNA was extracted from cells with Trizol (T9424, Merck) and reverse-transcribed into cDNA with a HiScript II first Strand cDNA Synthesis Kit (Vazyme, R212-01). To examine gene expression in cells, quantitative real-time PCR (qPCR) was carried out in triplicate with a ChamQ Universal SYBR qPCR Master Mix (Vazyme, Q711-02) as described previously (36). The mRNA levels of genes were normalized to those of GAPDH and presented as relative levels to control. Primers were designed using Primer 3 and synthesized by Sango Biotech. The primer sequences were as follows (5’ -3′): Hhatl, (forward: CAGAGCGGGTTTGTAACAGG) and (reverse: ATGATGGGCCCGAAGAAGAA); GRP78, (forward: AATGACCAGAATCGCCTGAC) and (reverse: CGCTCCTTGAGCTTTTTGTC); CHOP, (forward: TGGAAGCCTGGTATGAGGAC) and (reverse: AAGCAGGGTCAAGAGTGGTG); GAPDH, (forward: TGACAACGAATTTGGCTACA) and (reverse: GTGGTCCAGGGGTCTTACTC).

### Immunoblotting

Cells were collected and lysed in a buffer containing 1% NP-40, 150 mmol/L NaCl, 2 mM EDTA, 3% glycerol, and 50 mmol/L Tris (pH 7.5). Proteins were separated by SDS-PAGE and transferred onto polyvinylidene difluoride membranes (Millipore). The membranes were blocked in Tris-buffered saline containing 0.2% Tween 20 and 5% fat-free dry milk and incubated with primary antibodies and then horseradish peroxidase–conjugated secondary antibodies as described previously (37, 38). Specific proteins were visualized with enhanced chemiluminescence detection reagent according to the manufacturer’s instructions (New Cell & Molecular Biotech).

### Immunofluorescence microscopy

HeLa cells grown on glass coverslips were fixed with 4% paraformaldehyde for 30 min at room temperature followed by permeabilization in 0.5% Triton X-100/PBS for 20 min. Cells were then blocked with 2% bovine serine albumin in PBS and incubated with primary antibodies and fluorescein- or rhodamine-conjugated secondary antibodies (Proteintech) followed by staining with DAPI (Sigma-Aldrich). TUNEL BrightGreen apoptosis detection kit (Vazyme) was employed to examine apoptotic cells. Coverslips were then examined with PALM MicroBeam fluorescence microscope (Carl Zeiss, Inc).

### GST/MBP pull-down and immunoprecipitation

GST/MBP pull-down and immunoprecipitation were performed as described previously (35). For GST pull-down, the cell lysate was incubated with glutathione-conjugated agarose beads (Smart-Lifesciences, SA010010) at 4 °C for 2 h. For MBP pull-down, *in vitro*–translated Hhatl was incubated with bacterially purified MBP or MBP-LC3B immobilized on amylose-conjugated Dextrin Beads (Smart-Lifesciences, SA077005) at 4 °C for 2 h. The pull-down preparations were analyzed by immunoblotting. For immunoprecipitation, the cell lysate was incubated with anti-Flag affinity beads (Smart-Lifesciences, SA042001) at 4 °C for 2 h. The precipitated proteins were then examined by immunoblotting.

### Cell viability assays

Cell viability was determined using cell counting kit-8 (Cellcook, CT01A) according to the manufacturer’s protocol. Briefly, cells transfected with Flag-Hhatl or Hhatl siRNAs were seeded in 96-well plates and incubated with the cell counting kit-8 reagent for 2 h at 37 °C. The absorbance at 450 nm was measured using a microplate reader. Data are given from three independent experiments performed in technical triplicates.

### Apoptosis assay

The ratio of apoptotic cells was analyzed using the Annexin V-FITC/PI Apoptosis Detection Kit (Dojindo) according to the manufacturer’s instructions. Briefly, 1 to 5 × 10^5^ cells were harvested and washed with PBS. Cells were re-suspended and incubated in 400 μl binding buffer containing 5 μl Annexin V-FITC and 5 μl PI staining solution at room temperature in the dark for 20 min. The number of apoptotic cells was measured by flow cytometry using FACSCalibur Flow Cytometer (BD). Experiments were repeated in triplicate.

### Statistical analysis

Student’s *t* test was performed for the statistical analyses between two samples as appropriate using GraphPad Prism software. One-way ANOVA (followed by Tukey *post hoc* test) was performed to analyze differences among multiple groups. Data are demonstrated as mean ± SD from at least three biological replicates, and *p* < 0.05 was considered statistically significant.

## Data availability

The analyzed datasets generated during the study are available from the corresponding author on reasonable request.

## Supporting information

This article contains supporting information.

## Conflict of interest

The authors declare that they have no conflicts of interest with the contents of this article.
