# Supplementary figures and images for: Hhatl ameliorates endoplasmic reticulum stress through autophagy by associating with LC3
Source: J Biol Chem. 2024 May 4;300(6):107335. doi: 10.1016/j.jbc.2024.107335 (PMC11143907; doi:10.1016/j.jbc.2024.107335)

**A**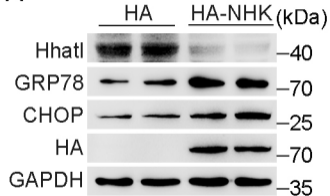**B**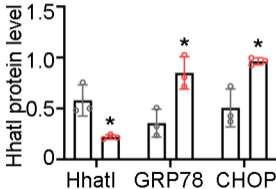

Supplement: Supporting Figure S1 [file mmc1.pdf]

A

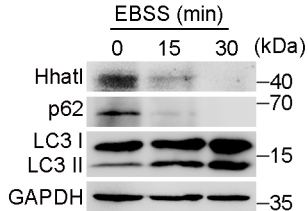

B

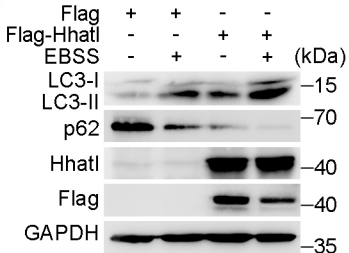

Supplement: Supporting Figure S2 [file mmc2.pdf]

**A**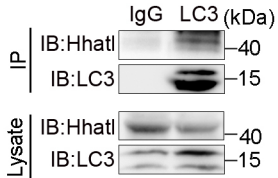**B**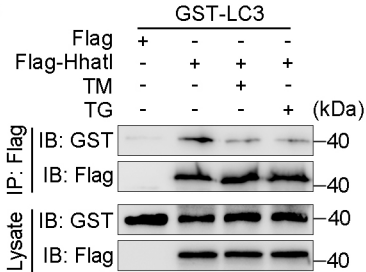

Supplement: Supporting Figure S3 [file mmc3.pdf]
