# Supplementary material for: Hhatl ameliorates endoplasmic reticulum stress through autophagy by associating with LC3
Source: J Biol Chem. 2024 May 4;300(6):107335. doi: 10.1016/j.jbc.2024.107335 (PMC11143907; doi:10.1016/j.jbc.2024.107335)
Supplement: Supporting Figures [file mmc5.docx]

**Figure S1. Hhatl expression is reduced under physiological ER stress.** *A*, HeLa cells were transfected with HA or HA-NHK for 24 hours. Cell lysates were subjected to immunoblotting, and probed with the indicated antibodies. *B*, Densitometric quantification for expression of Hhatl, GRP78 and CHOP was normalized to GAPDH protein levels. Data are presented as means ± SD (*n* = 3). Student’s t-test, **p* < 0.05.

**Figure S2. Hhatl promotes starvation-induced autophagy.** *A*, HeLa cells were treated with EBSS for indicated time periods. The cell lysates were subjected to immunoblot analysis, and probed with the indicated antibodies. *B*, HeLa cells transfected with Flag or Flag-Hhatl were treated with EBSS for nutrient starvation. Cell lysates were subjected to immunoblot analysis, and probed with the indicated antibodies.

**Figure S3. Hhatl interacts with the autophagic protein LC3.** *A*, Cell lysates were immunoprecipitated with control rabbit IgG or anti-LC3 antibody and probed with the indicated antibodies. *B*, the interaction between Hhatl and LC3 was decreased under ER stress. HEK293T cells were cotransfected with Flag vector or Flag-Hhatl and GST-LC3, and then treated with DMSO, TM or TG for 18 h. Cell lysates were subjected to immunoprecipitation with an anti-Flag antibody and probed with the indicated antibodies.
